# An inflammatory cytokine signature predicts IgA nephropathy severity and progression

**DOI:** 10.1002/mco2.783

**Published:** 2024-11-03

**Authors:** Lei Chen, Xizhao Chen, Guangyan Cai, Hongli Jiang, Xiangmei Chen, Min Zhang

**Affiliations:** ^1^ Department of Critical Care Nephrology and Blood Purification the First Affiliated Hospital of Xi'an Jiaotong University Xi'an Shaanxi China; ^2^ Department of Nephrology, First Medical Center of Chinese PLA General Hospital, Nephrology Institute of the Chinese People's Liberation Army, State Key Laboratory of Kidney Diseases, National Clinical Research Center for Kidney Diseases Beijing Key Laboratory of Kidney Disease Research Beijing China

**Keywords:** IgA nephropathy, Olink Proteomics, prognosis biomarkers, single‐cell sequencing

## Abstract

IgA nephropathy (IgAN) is the most prevalent primary glomerulonephritis, resulting in end‐stage renal disease and increased mortality rates. Prognostic biomarkers reflecting molecular mechanisms for effective IgAN management are urgently needed. Analysis of kidney single‐cell transcriptomic sequencing data demonstrated that IgAN expressed high‐expression levels of inflammatory cytokines TNFSF10, TNFSF12, CCL2, CXCL1, and CXCL12 than healthy controls (HCs). We also measured the urine proteins in 120 IgAN (57 stable and 63 progressive) and 32 HCs using the proximity extension assay (PEA), and the multivariable and least absolute shrinkage and selection operator (LASSO) logistic regression analysis both revealed that CXCL12, MCP1 were the prognostic significant variables to predict IgAN progression severity. These two proteins exhibited negative correlation with the estimated glomerular filtration rate (eGFR) and patients with higher expression levels of these two proteins had a higher probability to have poorer renal outcome. We further developed a risk index model utilizing CXCL12, MCP1, and baseline clinical indicators, which achieved an impressive area under the curve (AUC) of 0.896 for prediction of IgAN progression severity. Our study highlights the significance of the inflammatory protein biomarkers for noninvasive prediction of IgAN severity and progression, offering valuable insights for clinical management.

## Graphical abstract

We combined the single‐cell mRNA sequencing and proximity extension assay (PEA)‐based Olink urine protein profiling method to uncover the inflammation cytokines MCP1 and CXCL12 as potential noninvasive biomarkers to predict the IgAN severity and progression, offering valuable insights for clinical management.

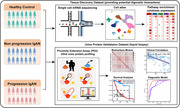

## 1 INTRODUCTION

IgA nephropathy (IgAN) stands as the prevailing form of glomerulonephritis on a global scale, accounting for a significant proportion of cases leading to kidney failure and chronic kidney disease (CKD).^1^, ^2^ Despite its initial description over half a century ago, our comprehension of the underlying pathophysiological mechanisms of this condition remains incomplete, and the quest for specific and efficacious treatments for IgAN continues to elude us.^3^ Furthermore, 30%–40% of IgAN individuals progress to end‐stage kidney disease within 20 years, presenting a formidable clinical management challenge.

IgAN is a common disease condition that is mostly caused by chronic inflammation. Its pathogenesis is influenced by several factors, including immunology, genetics, the environment, and nutrition.^4^, ^5^ Immunoglobulin‐containing immune complexes are constantly deposited in the glomerular mesangium in IgAN patients. This causes immunological damage and inflammatory reactions, which in turn cause podocyte damage, glomerulonephritis, mesangial cell (MC) proliferation, inflammatory cell recruitment, and myofibroblast formation.^6^, ^7^ Interstitial fibrosis, tubular atrophy, and glomerular sclerosis are the outcomes of the pathological interaction that these mediators of inflammation and fibrosis progressively create.^8^, ^9^ To improve the prognosis of IgAN patients, precise molecular targets for prevention and treatment are desperately needed.

In current clinical practice, the laboratory test indicators such as proteinuria, estimated glomerular filtration rate (eGFR) and complement C3 have been recommended as powerful and functional predictor of IgAN progression,^10^, ^11^, ^12^ was greatly limited by the sensitivity and specificity. Furthermore, several prognostic biomarker candidates screened by multiomics investigations have improved the prognosis accuracy of IgAN progression.^13^, ^14^, ^15^, ^16^, ^17^ Due to the cellular heterogeneity, it remains unclear what specific processes underlie the etiology and development of IgAN. The liquid biopsy such as plasma and urine protein biomarkers have been reported to improve patient outcomes and management, such noninvasive assessment of renal progression is critically required to prevent or delay kidney function decline in IgAN.

Single‐cell RNA sequencing (scRNA‐seq) is a powerful approach that provide valuable insights of cellular and molecular features within heterogenous biological tissues, which can help identify key cell players, pathogenic signaling pathway and potential intercellular crosstalk between cell types involved in disease progression.^18^, ^19^, ^20^, ^21^, ^22^, ^23^ Herein, by integration of multiomics data across three independent cohorts, we identified specific molecular features, and signaling pathways associated with IgAN progression. We also provided several noninvasive urine protein biomarkers to predict IgAN progression.

## 2 RESULTS

### 2.1 scRNA‐seq deciphers inflammation signature involved in human IgAN immune injury

**FIGURE 1:**
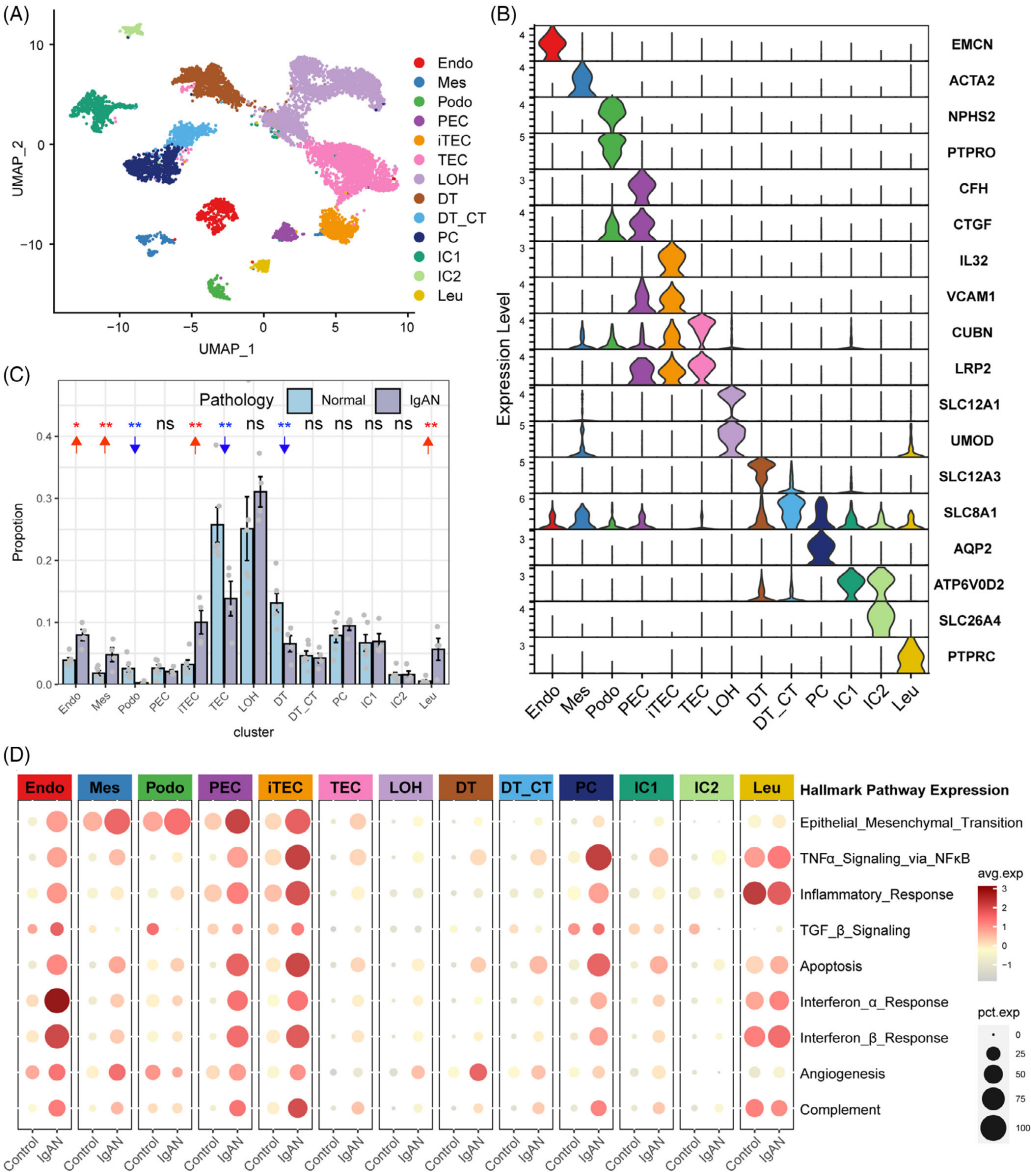
Comprehensive scRNA‐seq survey of the kidney ecosystem of IgA nephropathy (IgAN) and healthy control. (A) The cell identification from the scRNA‐seq survey of four IgAN samples and six control samples. (B) Violin plots showing the expression of representative marker genes of 13 distinct cell types. (C) The cell proportion of each cell type in distinct pathology samples. *p* values were assessed by Student's *t*‐test. (D) The expression level of hallmark inflammatory and fibrosis pathways in each cell type in distinct sample pathology.

**FIGURE 2:**
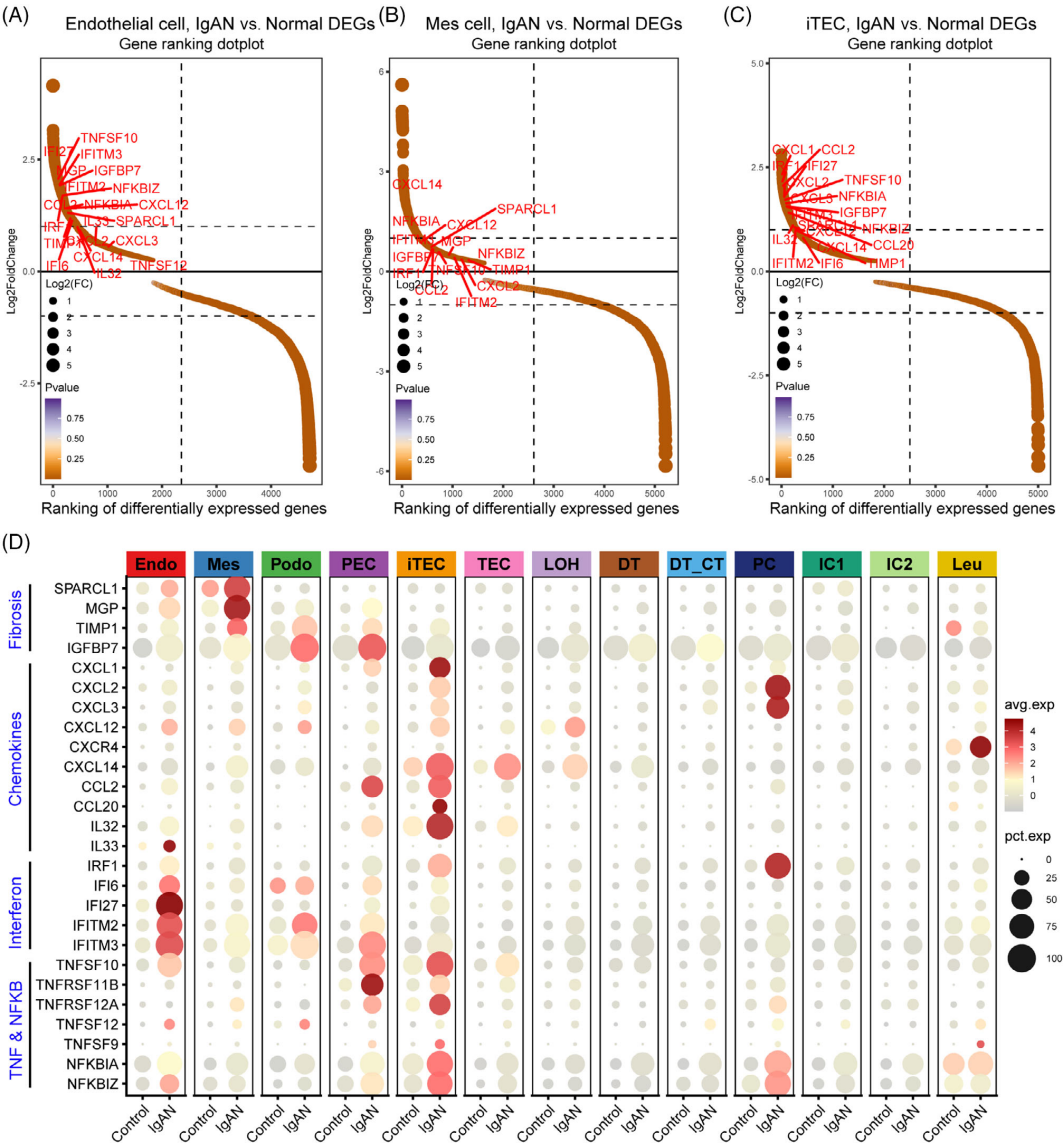
Comprehensive scRNA‐seq survey reveals the cell‐type‐specific signature expression difference in IgA nephropathy (IgAN) and healthy control (HC). (A) Gene ranking plot showing the upregulated expression of genes of endothelial cell of IgAN and health control. (B) Gene ranking plot showing the upregulated expression of genes of Mes cell of IgAN and health control. (C) Gene ranking plot showing the upregulated expression of genes of iTEC cell of IgAN and health control. (D) Dotplot showing the upregulated expression of genes of each cell type of IgAN and health control.

To explore the cellular and molecular features diversity of IgAN, we analyzed the scRNA‐seq data of IgAN and healthy control (HC) tissue samples. A total of 13 main cell subtypes were identified (Figure 1A and Table S1) based on the expression of classic marker genes (Figure 1B), top 10 differentially expressed genes (DEGs) and transcription factors (Figure S1). These subtypes included mesenchymal stromal cells (Mes, including fibroblast, pericyte, and MC, marked by ACTA2), endothelial cells (Endo, marked by EMCN), podocytes (Podo, marked by PTPRO), tubular epithelial cells (TECs, marked by LRP2), injured and inflammatory TEC (iTEC, marked by VACM1, and CUBN), parietal epithelial cells (PECs, marked by CFH), loop of Henle cells (LOH, marked by UMOD), distal tubular cells (DT, marked by SLC12A3), principal cells (PCs, marked by AQP2), intercalated cells (ICs, marked byATP6V1G3), leukocyte (Leu, marked by PTPRC). Cellular composition revealed that the proportion of Endo, Mes, iTEC, and Leu were elevated in IgAN patients, whereas Podo, the normal TEC, and DT were depleted compared with HC kidney tissues (Figure 1C, *p* < 0.05), and the cell portion distribution difference in IgAN and HC was validated by cell abundance scoring analysis using the IgAN bulk RNA‐seq dataset (Figure S2). Moreover, pathway enrichment analysis revealed that Endo, Mes, and iTEC derived from IgAN tissues showed significant enrichment of inflammation pathway such as tumor necrosis factor alpha (TNFα), interferon, inflammatory response, and complement, as well as fibrosis pathway such as epithelial–mesenchymal transition and transforming growth factor beta (TGF‐β; Figure 1D). Further cell‐type‐specific DEG analysis (Figures 2A–D and S3) revealed that Endo, Mes, and iTEC of IgAN had higher expression level of previously reported fibrosis markers (SPARCL1, MGP, TIMP1, and IGFBP7), inflammation markers belong to chemokines family (CXCL1/2/3, CXCL12, CXCL14, CCL2, and CCL20), TNFα signaling via NF‐κB pathway (TNFSF10, TNFSF12, NFKBIA, and NFKBIZ), and interferon family (IFI6, IFF27, and IFITM2/3) than that of HC. Interestingly, CXCR4, and TNFRSF12A which corresponded to the CXCL12, and TNFSF12‐associated receptor, were both highly expressed in Leu, Mes, and iTEC of IgAN (Figure S4). These results highlighted potential inflammatory ligand–receptor crosstalk involved in the immune injury of IgAN.

### 2.2 Identification of differentially expressed urine proteins associated with IgAN progression

To further investigate the role of inflammation pathways and biomarkers in classification and predicting progression of IgAN, we employed the Olink Proteomics to measure the urine proteins difference in 32 HCs as well as 57 stable IgAN samples and 63 progressive IgAN samples. The baseline and follow‐up characteristics of the 120 IgAN and 32 HC participants are summarized in Table 1. The progressive IgAN participants tended to have higher value of 24 h urine proteins, albumin‐to‐creatinine ratio (ACR), CKD stages, while with lower level of follow‐up eGFR value (*p* < 0.001).

**TABLE 1:** The baseline clinical features for IgA nephropathy (IgAN) patients and healthy control (HC).

	HC (*N* = 32)	Stable IgAN (*N* = 57)	Progressive IgAN (*N* = 63)	*p* value 1	*p* value 2
Age	43.7 (13.8)	44.5 (12.9)	40.8 (13.6)	0.293	0.164
Sex				0.594	0.853
Female	17 (53.1%)	24 (42.1%)	28 (44.4%)		
Male	15 (46.9%)	33 (57.9%)	35 (55.6%)		
Baseline eGFR (mL/min per 1.73 m^2^)	119 (13.4)	85.0 (22.1)	79.2 (16.2)	0.107	<0.001
Follow‐up eGFR (mL/min per 1.73 m^2^)	107 (13.0)	88.8 (27.2)	29.4 (18.3)	<0.001	<0.001
CKD stage				NA	<0.001
Stage 1	0 (0.00%)	40 (70.2%)	3 (4.76%)		
Stage 2	0 (0.00%)	10 (17.5%)	18 (28.6%)		
Stage 3	0 (0.00%)	4 (7.02%)	21 (33.3%)		
Stage 4	0 (0.00%)	2 (3.51%)	15 (23.8%)		
Stage 5	0 (0.00%)	1 (1.75%)	6 (9.52%)		
Hypertension				0.008	0.005
With	20 (62.5%)	30 (52.6%)	50 (79.4%)		
Without	12 (37.5%)	27 (47.4%)	13 (20.6%)		
Baseline urine protein (g/24 h)	0.03 (0.04)	0.84 (0.30)	2.38 (1.20)	<0.001	<0.001
ACR (mg/g)	15.2 (6.94)	95.7 (49.6)	172 (85.9)	<0.001	<0.001

Abbreviations: ACR, albumin‐to‐creatinine ratio; CKD, chronic kidney disease; eGFR, estimated glomerular filtration rate; follow‐up eGFR, the eGFR value at the last follow‐up time point during the study.

CKD stages were divided by eGFR value ≥90, =60–89, =30–59, =15–29, <15 mL/min per 1.73 m^2^, respectively, according to the Kidney Disease Outcomes Quality Initiative (KDOQI).

*p* value 1: The overall *p* value between progressive IgAN, stable IgAN, and HC.

*p* value 2: The overall *p* value between progressive IgAN, and stable IgAN.

**FIGURE 3:**
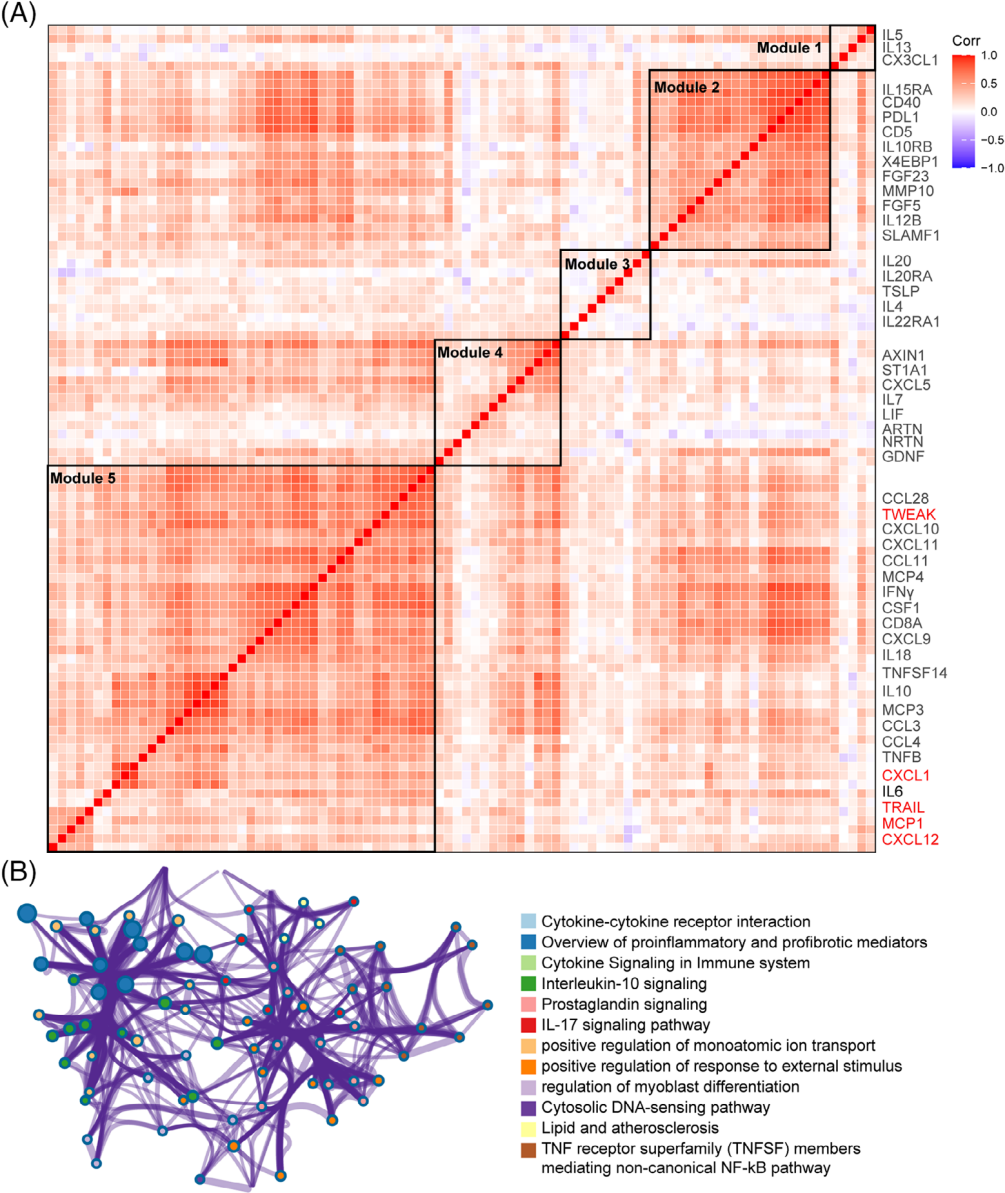
Identification of IgA nephropathy (IgAN)‐associated urine hub proteins. (A) The pairwise correlations among urine proteins in IgAN patients. Each row and column represent one of the 180 IgAN‐associated urine proteins. Red and blue squares indicate positive and negative correlations between protein pairs, respectively. Black squares denote the five protein modules based on hierarchical clustering, and numbers in brackets on the right indicate the cluster number. (B) Enrichment of the Gene Ontology (GO) terms in the proteins pair associated with module 5.

**FIGURE 4:**
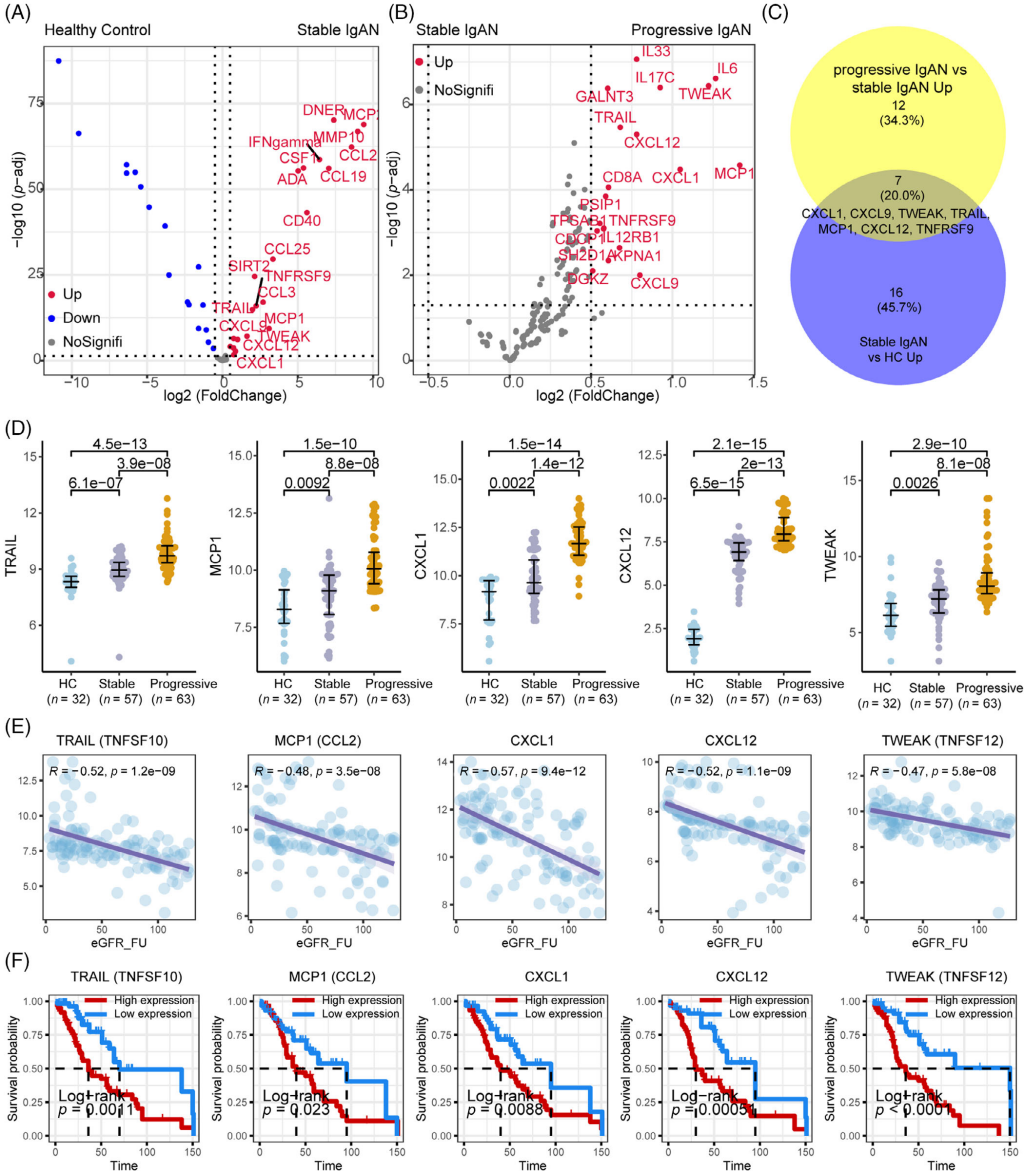
Identification of IgA nephropathy (IgAN) progression‐associated urine proteins. (A) Volcano plot showing the differentially expressed urine proteins between stable IgAN and healthy control (HC). The red dots indicate urine proteins in stable IgAN patients were upregulated compared to HC. (B) Volcano plot showing the differentially expressed urine proteins between stable IgAN and progressive IgAN. The red dots indicate urine proteins in progression IgAN patients were upregulated compared to stable IgAN. (C) Venn diagram showing the intersection proteins upregulated both in two compare groups (stable IgAN vs. HC, and progressive IgAN vs. stable IgAN). (D) Beeswarm showing the expression of CXCL1, CXCL12, TWEAK, TRAIL, and MCP1 in HC, stable, and progressive IgAN. (E) The scatterplot showing the correlations of the urine proteins CXCL1, CXCL12, TWEAK, TRAIL, and MCP1 with the follow‐up eGFR value. (F) The Kaplan–Meier renal survival curves of patients with IgAN according to the urine proteins CXCL1, CXCL12, TWEAK, TRAIL, and MCP1.

After data control filtering, we then carried out the correlation network analysis (Figure 3A) to investigate the coregulation patterns of the 88 immune response and 92 inflammatory urine proteins, which produced five unique urine expression modules. The module 5 comprises 22 upregulated proteins (et., al TWEAK, CXCL1, MCP1, TRAIL, CXCL9, CXCL10, IL10, IL18, and CXCL12) involved in cytokine–cytokine receptor interaction, proinflammatory and profibrotic pathways, IL‐17 signaling pathway and TNF receptor superfamily (TNFSF) members mediating noncanonical NF‐kB pathway (Figure 3B). Among the 180 measured urine proteins, 22 urine proteins were upregulated in IgAN compared with HCs (Figure 4A) and 19 urine proteins were upregulated in progressive IgAN compared with stable IgAN patients (Figure 4B), of which seven proteins including TWEAK, CXCL1, MCP1, TRAIL, and CXCL12 were significantly upregulated in IgAN, with higher expression level in progressive IgAN (*p* < 0.05 and abs (logFC) > 0.5; Figure 4C,D). Additionally, the inflammation cytokines TWEAK, CXCL1, MCP1, TRAIL, and CXCL12 showed both significant negative association with the follow‐up eGFR value, while significant positive correlation with the ACR and 24 h urine protein (Figures 4E and S5, *p* < 0.001). By analyzing the public Nephroseq dataset (https://nephroseq.org), we found CXCL12, CCL2 (MCP1), and CXCL1 expression was negatively associated with the eGFR value, while positively correlated with the 24 h urine protein, and urine protein–creatinine ratio, these results further validated our findings (Figure S6). Finally, the renal survival study revealed that IgAN patients tended to have poorer outcomes (30% eGFR drop, Figure 4F) when their expression levels of TWEAK, CXCL1, MCP1, TRAIL, and CXCL12 were greater.

### 2.3 Correlation between the urine inflammation proteins with severity of IgAN

Have identified that the urine inflammation proteins TWEAK, CXCL1, MCP1, TRAIL, and CXCL12 as a potential valuable biomarker of IgAN progression. We further performed correlation analysis of five urine inflammation proteins TWEAK, CXCL1, MCP1, TRAIL, and CXCL12 expression levels with the baseline clinical and histologic manifestations. All the five urine inflammation proteins showed good relationships with CKD stages, ACR value, 24 h urine proteins, and Oxford T and scores (Figure S5 and Table 2). The expression of CXCL1, TWEAK, and CXCL12 was higher in patients with severe CKD stages, Oxford T scores, and extracapillary proliferation (Ep) lesions score than in patients with mild CKD stages, Oxford T scores, and without tubular and interstitial lesions; however, patients with varying Oxford M, and S scores showed similar levels of TWEAK, CXCL1, MCP1, TRAIL, and CXCL12 (Table 2).

**TABLE 2:** Correlation between the urine TWEAK, CXCL1, CXCL12, MCP1, and TRAIL protein with severity of IgA nephropathy.

	CXCL1		CXCL12		MCP1		TRAIL		TWEAK	
Characteristics	Expression	*p* value	Expression	*p* value	Expression	*p* value	Expression	*p* value	Expression	*p* value
Hypertension		0.009		0.029		0.0232		0.045		0.003
With	11.2 (1.36)		7.68 (1.10)		9.86 (1.28)		9.57 (0.87)		8.10 (1.84)	
Without	10.3 (1.66)		7.19 (1.14)		9.43 (1.46)		9.16 (1.09)		7.26 (1.18)	
CKD stage		<0.001		<0.001		<0.001		<0.001		<0.001
1	10.1 (1.47)		6.64 (1.04)		8.99 (1.31)		8.88 (1.05)		6.66 (1.18)	
2	11.0 (1.35)		7.89 (0.94)		9.67 (1.07)		9.49 (0.62)		7.93 (1.00)	
3	11.2 (1.45)		8.04 (0.90)		9.82 (1.47)		9.61 (0.88)		8.65 (1.87)	
4	11.8 (1.10)		8.03 (0.79)		10.8 (1.00)		10.3 (0.63)		8.81 (1.57)	
5	11.8 (1.24)		8.12 (0.78)		10.2 (1.92)		9.97 (0.56)		9.03 (2.21)	
Mesangial hypercellularity (M) score		0.661		0.065		0.829		0.181		0.305
0	11.0 (1.63)		7.27 (1.18)		9.69 (1.41)		9.30 (0.72)		7.64 (1.33)	
1	10.8 (1.43)		7.67 (1.07)		9.63 (1.42)		9.53 (1.09)		7.94 (1.89)	
Endocapillary proliferation (E) score		0.748		0.024		0.335		0.037		0.043
0	10.8 (1.69)		7.23 (1.15)		9.50 (1.38)		9.23 (0.72)		7.47 (1.34)	
1	10.9 (1.38)		7.71 (1.08)		9.76 (1.43)		9.58 (1.08)		8.07 (1.87)	
Segmental glomerulosclerosis/adhesion (S) score		0.764		0.343		0.754		0.152		0.293
0	10.9 (1.72)		7.38 (1.11)		9.60 (1.39)		9.28 (0.72)		7.61 (1.35)	
1	10.9 (1.40)		7.59 (1.14)		9.68 (1.42)		9.52 (1.06)		7.93 (1.84)	
Tubular atrophy/interstitial fibrosis (T) score		0.006		<0.001		0.031		0.001		<0.001
0	10.5 (1.61)		7.09 (1.10)		9.36 (1.39)		9.21 (0.68)		7.35 (1.41)	
1	11.3 (1.24)		7.95 (0.96)		9.92 (1.38)		9.56 (1.22)		8.13 (1.81)	
2	11.5 (1.28)		8.28 (0.87)		10.3 (1.30)		10.3 (0.70)		9.37 (1.65)	
Extracapillary proliferation (Ep) score		0.066		<0.001		0.028		0.044		0.044
0	10.6 (1.65)		7.09 (1.18)		9.46 (1.41)		9.25 (0.69)		7.49 (1.36)	
1	11.1 (1.34)		7.88 (0.95)		9.92 (1.39)		9.60 (1.13)		8.11 (1.90)	

### 2.4 Screening the significant prognostic variables to predict IgAN progression

To screen the significant prognostic variables to predict IgAN progression, we first performed univariate logistic regression analysis and the results suggested that TWEAK, CXCL1, MCP1, TRAIL, and CXCL12 as a potential valuable predictor in IgAN progression (unadjusted analysis: CXCL1: hazard ratio [HR], 1.21; 95% confidence interval [95% CI], 1.08–1.36; *p *= 0.001; TRAIL: HR, 1.84; 95% CI, 1.5–2.26; *p* < 0.001; MCP1: HR, 1.93; 95% CI, 1.5–2.48; *p* < 0.001; CXCL12: HR, 3; 95% CI, 2.09–4.3; *p* < 0.001; TWEAK: HR, 1.86; 95% CI, 1.56–2.2; *p* < 0.001, Table 3). After multivariable adjustment, there was a strong correlation found between the increasing severity of IgAN progression and increased levels of urine MCP1 and CXCL12. (CXCL12: HR, 1.79; 95% CI, 1.38–2.33; *p* < 0.001; MCP1: HR, 2.14; 95% CI, 1.59–2.89; *p* < 0.001, Table 3). Among the clinical indicators, both the univariate and multivariable logistic regression showed that Oxford classification T lesion and extracapillary proliferation (Ep) lesion were the most prognostic significant risk factors to predict the IgAN progression (Table 3).

**TABLE 3:** Logistic regression model of risk factors associated with IgA nephropathy (IgAN) patients’ progression.

	Univariable logistic regression		Multivariable logistic regression	
Characteristics	HR (95% CI)	*p* value	HR (95% CI)	*p* value
CXCL1	1.21 (1.08–1.36)	0.001	1.52 (0.96–2.41)	0.075
CXCL12	3 (2.09–4.3)	<0.001	1.79 (1.38–2.33)	<0.001
TRAIL	1.84 (1.5–2.26)	<0.001	1.06 (0.72–1.57)	0.757
MCP1	1.93 (1.5–2.48)	<0.001	2.14 (1.59–2.89)	<0.001
TWEAK	1.86 (1.56–2.2)	<0.001	0.93 (0.79–1.1)	0.393
ACR	1.01 (1–1.01)	<0.001	1 (1–1)	0.421
Baseline 24 h urine protein	1.84 (1.51–2.24)	<0.001	1.43 (1.15–1.8)	0.002
**Oxford classification**				
M score	0.96 (0.56–1.66)	0.897	0.97 (0.83–1.13)	0.68
E score	1.46 (0.83–2.58)	0.189	3 (0.32–28.59)	0.339
S score	1.19 (0.67–2.12)	0.557	0.95 (0.1–8.8)	0.963
T score	2.02 (1.42–2.87)	<0.001	1.75 (1.11–2.76)	0.016
Ep score	2.23 (1.26–3.96)	0.006	6.13 (1.37–27.31)	0.017

Abbreviations: ACR, albumin‐to‐creatinine ratio; CI, confidence interval; HR, hazard ratio.

**FIGURE 5:**
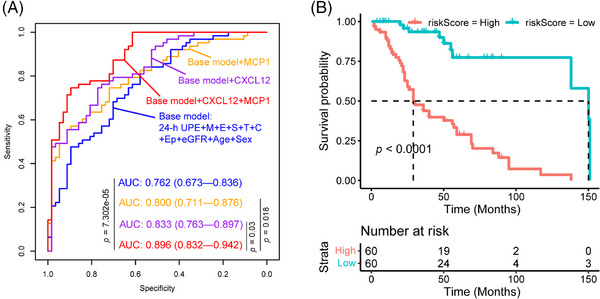
The diagnostic power for prediction of IgA nephropathy (IgAN) progression using the cytokines biomarkers classifier and clinical indicators. (A) The diagnostic power of base model (24 h urine protein expression [24‐h UPE] value, age, sex, estimated glomerular filtration rate [eGFR], Oxford classification MEST lesion, and extracapillary proliferation [Ep] lesion), base model + CXCL12, base model + MCP1, and base model + CXCL12 + MCP1 for the prediction of IgAN progression. (B) The Kaplan–Meier renal survival curves of patients with IgAN according to the risk index model (combination of clinical indicators, MCP1 and CXCL12) score.

We further employed the least absolute shrinkage and selection operator (LASSO) logistic regression analysis to rigorously screen the significant prognostic urine protein biomarker variables for prediction of IgAN severity. Consistent with multivariate regression analysis, LASSO logistic regression analysis also showed that CXCL12, MCP1 were the significant urine protein biomarkers variables for prediction of IgAN progression. We therefore employed the baseline clinical indicators (24 h urine protein expression [24‐h UPE], age, sex, eGFR, Oxford classification MEST, and extracapillary proliferation [Ep] lesion) to generate the base model for classification of progressive and stable IgAN patients. We also developed three models (Figure 5A) using the clinical indicators and urine proteins: base model + CXCL12, base model + MCP1, and base model + CXCL12 + MCP1. Our result (Figure 5A) showed that the combination of clinical indicators, MCP1 and CXCL12 had the best performance to predict the progression of IgAN compared with base model (*p* = 7.302e‐05), base model + CXCL12 (*p* = 0.03), and base model + MCP1 (*p* = 0.018), respectively. When we selected 30% eGFR value decline as composite endpoint of IgAN during the follow‐up, the risk index model (combination of clinical indicators, MCP1 and CXCL12) score was correlated with poor prognosis of IgAN (Figure 5B).

## 3 DISCUSSIONS

IgAN is a prototypical chronic inflammation mediated disease, with inflammation playing a pivotal role in its pathogenesis and progression. Numerous circulating protein biomarkers^13^, ^14^, ^15^, ^16^, ^17^ have been reported to predict the severity and progression of IgAN. However, the mechanistic underpinnings explaining these associations have remained largely unexplored. In this comprehensive study, we systematically evaluated candidate inflammation biomarkers in relation to IgAN severity, utilizing an unbiased scRNA‐seq survey of the IgAN microenvironment. Our investigation unveiled that glomerular endothelial cells (Endo), mesenchymal cells (Mes), and proinflammatory tubular epithelial cells (iTECs) in IgAN were characterized by significantly elevated expression levels of proinflammatory and profibrotic signatures. These findings were accompanied by the activation of multiple inflammatory pathways, including inflammatory response, interferon response, complement, TNFα signaling via NF‐κB pathway, and cytokine–cytokine receptor interaction pathways, compared to HCs. As we know, tubular injury, activation of mesenchymal cells, and endothelial cells could lead to kidney fibrosis, and CKD progression.^24^ Furthermore, we validated an inflammation protein panel, including TWEAK (TNFSF12), CXCL1, MCP1, TRAIL, and CXCL12, using proximity extension assay (PEA)‐based urine proteomic assays.^25^, ^26^ This panel significantly improved the prediction of kidney outcomes in IgAN patients. Notably, our analysis identified CXCL12‐CXCR4 and TNFSF12‐TNFRSF12A as the prominent ligand–receptor pairs involved in the cellular crosstalk within IgAN, potentially linked to immune injury. Importantly, our renal survival analysis and ordinal logistic regression provided robust evidence that TWEAK (TNFSF12), CXCL1, MCP1, TRAIL, and CXCL12 could serve as noninvasive biomarkers for predicting IgAN progression.

Among the key biomarkers, CXCL1 plays a multifaceted role in inflammation, angiogenesis, tumorigenesis, and wound healing. It serves as a potent chemoattractant for neutrophils, predominantly via CXCR2 receptors. Our scRNA‐seq data, along with recent studies in acute kidney injury,^27^ supported the notion that CXCL1 is highly expressed by profibrotic and inflammatory TECs, thereby contributing to inflammation in kidney disease and promoting fibrosis.

CXCL12 (SDF1) induces migration and activation of leukocytes through interaction with receptor CXCR4. CXCL12 can also play an inflammatory role, has been demonstrated as being involved in tissue fibrosis.^28^ Increasing evidences showed that CXCL12 participated in the progression of various kidney diseases, such as AKI, diabetic kidney disease, and lupus nephritis.^29^ In our study, the scRNA‐seq survey showed that CXCL12 was highly expressed endothelial cells, iTEC, and Mes, with higher expression level in that of IgAN compared to HC. We also demonstrated that the CXCL12‐CXCR4 chemokine–chemokine interaction pathway was activated in IgAN. Urinary CXCL12 protein levels were significantly associated with declining renal eGFR and adverse renal outcomes, underscoring its importance as a predictor of IgAN progression.

CCL2 contribute to inflammatory and fibrotic process^30^ by supporting leukocytes recruitment, and activation of angiogenesis, fibroblast collagen synthesis, myofibroblast differentiation. Our work demonstrated that MCP1 (CCL2) protein is increased in progressor IgAN patients’ urine samples, which was significantly associated with renal eGFR decline and worse renal outcome in IgAN. The scRNA‐seq data showed that iTECs, endothelial cells, and PEC expressed high level of CCL2, with higher expression level in progressive IgAN patients. Recent studies have shown that baseline urine CCL2 levels are good predictors of the risk of progression to a variety of kidney diseases.^31^, ^32^ Hence, CCL2 is one of the potential markers for liquid biopsy to predict kidney diseases progression. It is worth mentioning that such studies have also pointed out that CCL2 in combination with other biomarkers have more predictive value than itself alone. In our study, CCL2 combined with CXCL12 showed best performance to predict the progression of IgAN. In fact, a series of studies showed that these two respective chemokine targets mediate different pathomechanisms of kidney diseases and dual blockade of CCL2 and CXCL12 leads to additive effects on the in prevention of progression of nephropathy and proliferative lupus nephritis.^29^, ^33^ Although the effects of dual blockade of this two chemokine have not been tested in IgAN, our results indicate it would be a possibility worth trying.

Ligands and receptors of the TNF superfamily regulate immune responses and homeostatic functions with potential diagnostic and therapeutic implications. The TNF superfamily member TNFSF12 is known as TNF‐like weak inducer of apoptosis (TWEAK^34^), it was shown that acts through its receptor, Fn14 (TNFRSF12A), to promote proinflammatory responses in kidney cells, including the production of MCP‐1, RANTES, IP‐10, and KC. Activation of TWEAK/Fn14 pathway results in kidney disease^35^, ^36^ progression by promoting MC proliferation, vascular endothelial cell activation, and renal cell death. Inhibition of TWEAK/Fn14 axis^37^ showed significant influence to protect against acute liver failure^38^ and liver fibrosis.^39^ TNF‐related apoptosis‐inducing ligand (TRAIL, TNFSF10) is a cytokine belonging to the TNF superfamily that has been recently linked to the pathogenesis of diabetic nephropathy and lupus nephritis.^40^ In our study, we demonstrated the first time that TWEAK/Fn14 axis was activated in IgAN ecosystem by scRNA‐seq analysis, and the PEA‐based urine proteomics revealed that TNF superfamily members TWEAK and TRAIL as prognostic markers for prediction of IgAN progression.

Our research has a number of other drawbacks. We were able to determine the key cell‐specific feature gene, pathway, and ligand–receptor pairs involved in the IgAN ecosystem by an unbiased scRNA‐seq survey, and we found statistically significant correlations between protein biomarkers and IgAN progression and clinical outcomes. However, we are unable to determine whether the proteins studied here are IgAN‐specific progression biomarkers or are synthesized by other organs or in other clinical conditions. Second, the reduced sample size hampered our proteomic results, which need further comprehensive validation by multicenter cohorts. In summary, we found that the inflammatory proteins CXCL1, TWEAK, TRAIL, MCP1, and CXCL12 are prospective predictors of IgAN progression by the use of an unbiased proteomic method and high‐resolution scRNA‐seq survey. An underlying molecular architecture linking urine proteomics to TWEAK‐Fn14 and CXCL12‐CXCR4 signaling and the advancement of illness in IgAN patients was identified using integrative bioinformatics research. These biomarkers might help with prognosis estimation and clinical decision making, and they could be employed in research to produce noninvasive assessments of the degree of advancement of IgAN.

## 4 MATERIALS AND METHODS

### 4.1 Study design and participants

This retrospective urine proteomic study was conducted at the First Medical Center of the PLA General Hospital, aimed to investigate the differentially expressed urinary proteomic profiles of progressive IgAN, stable IgAN, and HC donors. A total of 120 individuals diagnosed with IgAN and 32 HC donors were enrolled using the propensity score matching (PSM) method, the 120 IgAN patients has a median of 62‐month regular follow‐up period. Table 1 displays the patients' comprehensive clinical characteristics. The diagnosis of IgAN was established based on the presence of dominant IgA deposition in the mesangium, as confirmed by immunofluorescence, light microscopy, and electronic microscopy. After comprehensive clinical and laboratory evaluations, patients with concurrent Henoch–Schönlein purpura, liver cirrhosis, or other secondary etiologies of IgAN were eliminated. IgAN patients with 24 h urine protein >1 g were defined as high‐risk progression cases.^41^, ^42^ Patients were treated according to the Kidney Disease Improving Global Outcomes (KDIGO) recommendations for the duration of the follow‐up period,^43^ an eGFR decrease of more than 30% or incident ESKD during a 5‐year follow‐up period was considered a renal outcome.

### 4.2 scRNA‐seq data processing

We obtained the single‐cell gene expression data of human IgAN and HC samples from the Gene Expression Omnibus (GEO) website (accession numbers GSE171314,^44^ GSE151302,^45^ respectively). Data analysis was performed using the Seurat^46^ package (version 4.0.0) with standard protocols as previously reported.^18^, ^19^ Cells deemed low quality were removed if they contained more than 50% of unique molecular identifiers (UMIs) generated from the mitochondrial genome or less than 2001 UMIs. The SeuratWrappers package with the RunFastMNN function was used to integrate single‐cell transcriptome expression profiles across samples, and the DoubletFinder R package was utilized to address potential cell doublets.^47^ Highly variable genes were selected for Principal Component Analysis (PCA), and the top 30 significant principal components were used for UMAP dimension reduction and visual representation of gene expression patterns. Cell types were defined according to known classic marker genes and top 10 DEGs.

### 4.3 Pathway analysis and cell subtype abundance score in bulk RNA‐seq samples

We employed Gene Set Variation Analysis (GSVA)^48^ to assess the pathway enrichment in scRNA‐seq cell types derived from IgAN and HC using the hallmark gene sets from the Molecular Signatures Database (MSigDB). The human IgAN and HC bulk‐seq data were used to validate the scRNA‐seq findings, which can be accessed from GEO website with identifier GSE93798.^49^ We employed the GSVA package to assess the relative cell subtypes abundance in bulk RNA‐seq data of IgAN.

### 4.4 PEA platform‐based urine proteomics

We collected urine samples at a median time point of 6.2 h before the biopsy, with an interquartile range (IQR) spanning from 1.6 to 26.7 h. These urine specimens were processed and stored at −80°C. Subsequent proteomic and biomarker measurements were conducted on urine samples. We worked with Olink Proteomics to do urine proteome analysis, utilizing two manufacturer‐validated, commercially available panels called “inflammation” (version 3021) and “immune response” (version 3203). For our investigation, we concentrated on 180 nonoverlapping proteins out of the 184 total proteins included in these two panels. To reduce the possibility of cross‐reactivity, two PEA^25^, ^26^ probes—monoclonal or polyclonal antibodies—that are oligonucleotide‐labeled bind to each target protein separately in this test. Complementary probes for each target protein hybridize and expand upon engagement, creating a distinct sequence that permits digital protein identification. These sequencing operations were performed on an Illumina NovaSeq 6000 machine. Normalized Protein eXpression (NPX) units, which were logarithmically scaled (log2) and obtained from count (Ct) values, were converted from the quantifiable quantities of known sequences.

### 4.5 Renal survival analysis according to the urine inflammatory cytokines expression

The UPE data and follow‐up data of 57 stable IgAN samples and 63 progressive IgAN samples were used to evaluate the prognostic value of the inflammatory cytokine's signatures identified by unbiased scRNA‐seq analysis. Our objective was to evaluate the predictive power of these inflammatory cytokine signatures in the context of IgAN prognosis. For the renal survival study, we used a 30% drop in eGFR as the endpoint of IgAN development.^15^, ^50^ To stratify IgAN patients, we categorized them into high‐ and low‐expression group based on the median expression levels of five inflammatory cytokines. Subsequently, we generated Kaplan–Meier renal survival curves using the R package “survminer.”

### 4.6 Screening the significant prognostic variables to predict IgAN progression

Two approaches were used to screen the prognostic important urine protein biomarker factors to predict the development of IgAN: the LASSO COX logistic regression analysis and the cox multivariable logistic regression analysis. In summary, multivariate COX regression analysis was carried out based on the features that were shown to be substantially linked with IgAN advancement by univariate COX, in order to screen the prognostic important factors. In order to conduct the LASSO COX logistic regression analysis, we also used 15 variables, which included 10 clinical indicators and five inflammation proteins. These variables passed the screening process for the Cox multivariable logistic regression analysis, and the results of the LASSO COX logistic regression analysis were retained for building the IgAN progression model.

### 4.7 Bioinformatics and statistics analysis

The significantly upregulated or downregulated urine proteins in two sample groups were determined using unpaired Welch's *t*‐test implemented in statistical software environment R (version 3.3.2) and screened by heat map and volcano plot analysis using R package ComplexHeatmap^51^ and ggplot2, respectively. The urine proteins with a *p* value <0.05 and a fold change >0.5 between IgAN and HC, between progressive IgAN and stable IgAN samples were considered significant. The Metascape^52^ online website (https://metascape.org/gp/index.html) was used for pathway and Gene Ontology (GO) function enrichment analysis. The HR was estimated using the Cox proportional hazard model. In the multivariate Cox regression model, the *p* value ≤0.05 in the univariate Cox regression. All the *p* values were two‐sided, and the *p* values ≤0.05 were considered statistically significant. All statistical analyses were performed using the statistical software R (version 4.1.0) and GraphPad Prism (version 9.0).

## AUTHOR CONTRIBUTIONS

Min Zhang, Xiangmei Chen, and Hongli Jiang conceptualized and designed the study. Xizhao Chen was responsible for the collection of the biopsies, the follow‐up of the patients. Min Zhang analyzed the data with the help of Lei Chen, Xizhao Chen was responsible for sample preparation and data quality control. Min Zhang, Lei Chen, and Xizhao Chen drafted the manuscript. Xiangmei Chen and Min Zhang critically revised the manuscript for important intellectual content. Min Zhang, Hongli Jiang, and Xiangmei Chen obtained funding and supervised the study. All the authors participated in the discussion. All the authors have read and approved the final manuscript.

## CONFLICT OF INTEREST STATEMENT

The authors declare no conflicts of interest.

## ETHICS STATEMENT

The study protocol was subjected to rigorous review and approval by the Ethics Committee of the First Medical Center of the PLA General Hospital (Approval ID: S2015‐061‐01). Written informed consent was obtained from each participant, affirming their voluntary participation, and understanding of the study's objectives and procedures.

## Supporting information



SUPPORTING INFORMATION

SUPPORTING INFORMATION

## Data Availability

The urine proteomic data of 32 HC donors and 120 IgAN patients using the PEA‐based Olink Proteomics platform have been uploaded in Zenodo with identifiehttps://doi.org/10.5281/zenodo.8358543. Other public published single‐cell RNA sequencing data of human IgAN and HC can be accessed from the GEO website (accession numbers GSE171314,44 GSE151302,45 respectively).
