# Supplementary material for: An inflammatory cytokine signature predicts IgA nephropathy severity and progression
Source: MedComm (2020). 2024 Nov 3;5(11):e783. doi: 10.1002/mco2.783 (PMC11531656; doi:10.1002/mco2.783)
Supplement: Supplementary file 1 — SUPPORTING INFORMATION [file MCO2-5-e783-s001.docx]

Title: An inflammatory cytokine signature predicts IgA nephropathy severity and progression

Authors: Lei Chen^1,#^, Xizhao Chen^2,#^, Guangyan Cai^2^, Hongli Jiang^1,*^, Xiangmei Chen^2,*^, Min Zhang^2,*^

Affiliations:

^1^Department of Critical Care Nephrology and Blood Purification, the First Affiliated Hospital of Xi'an Jiaotong University. Xi'an, Shaanxi, 710061, China.

^2^Department of Nephrology, First Medical Center of Chinese PLA General Hospital, Nephrology Institute of the Chinese People's Liberation Army, State Key Laboratory of Kidney Diseases, National Clinical Research Center for Kidney Diseases, Beijing Key Laboratory of Kidney Disease Research, Beijing 100853, China.

Supplemental materials

Supplementary Table S1: The top 30 differentially expressed genes in cell types identified by scRNA-seq.

Supplementary figures and legends:


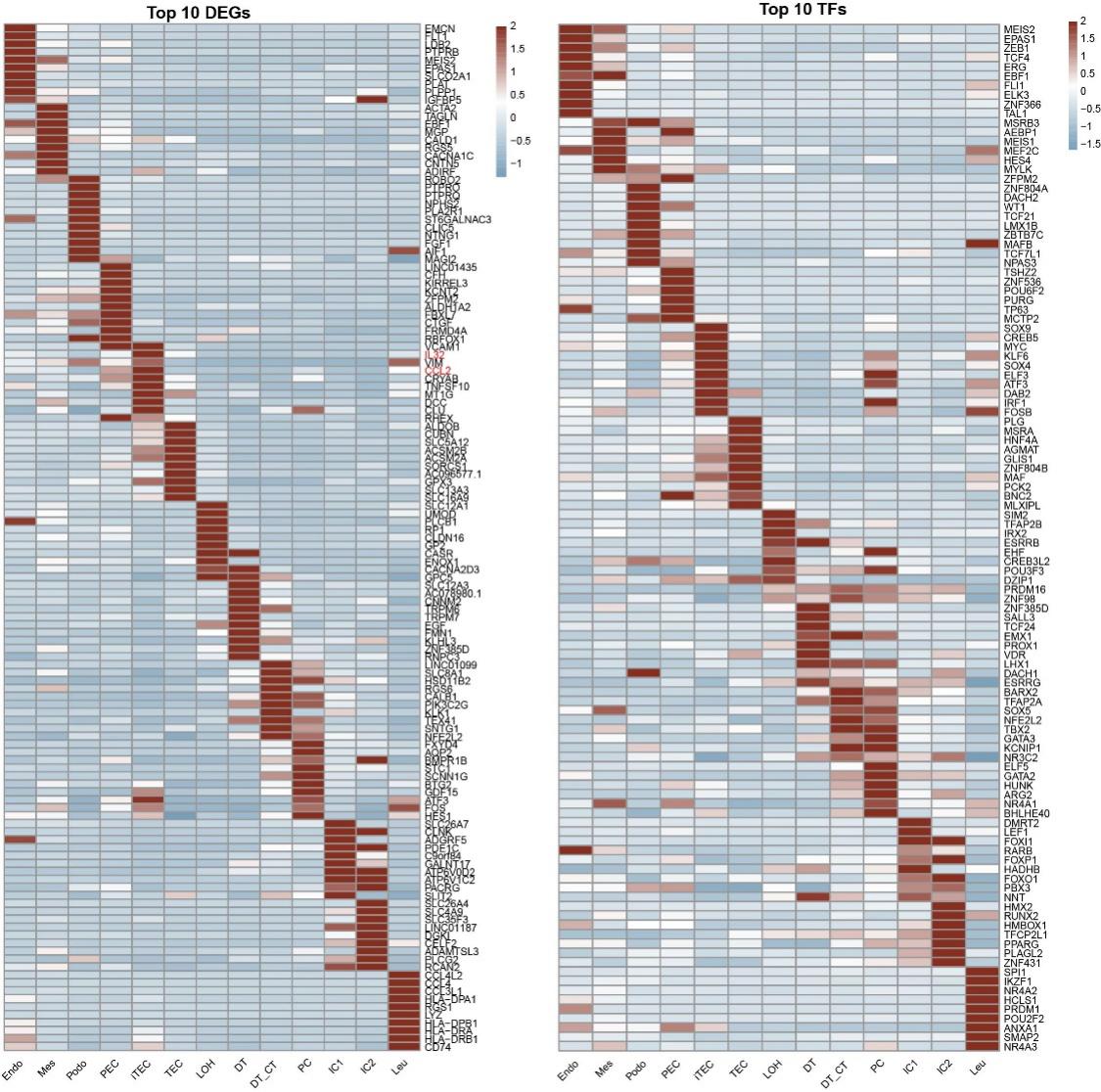


Figure S1: Heatmap showing the expression of top differentially expressed genes (left) and transcription factors (right) for 13 distinct cell types.


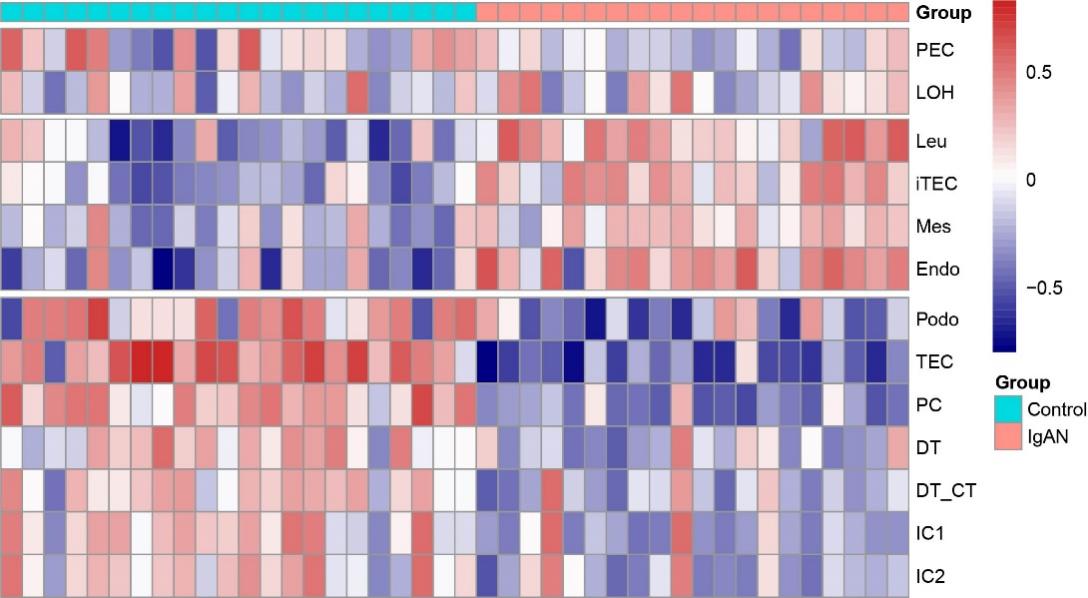


Figure S2: Heatmap showing the relative cell type abundance of IgAN and healthy control, estimated by GSVA analysis.


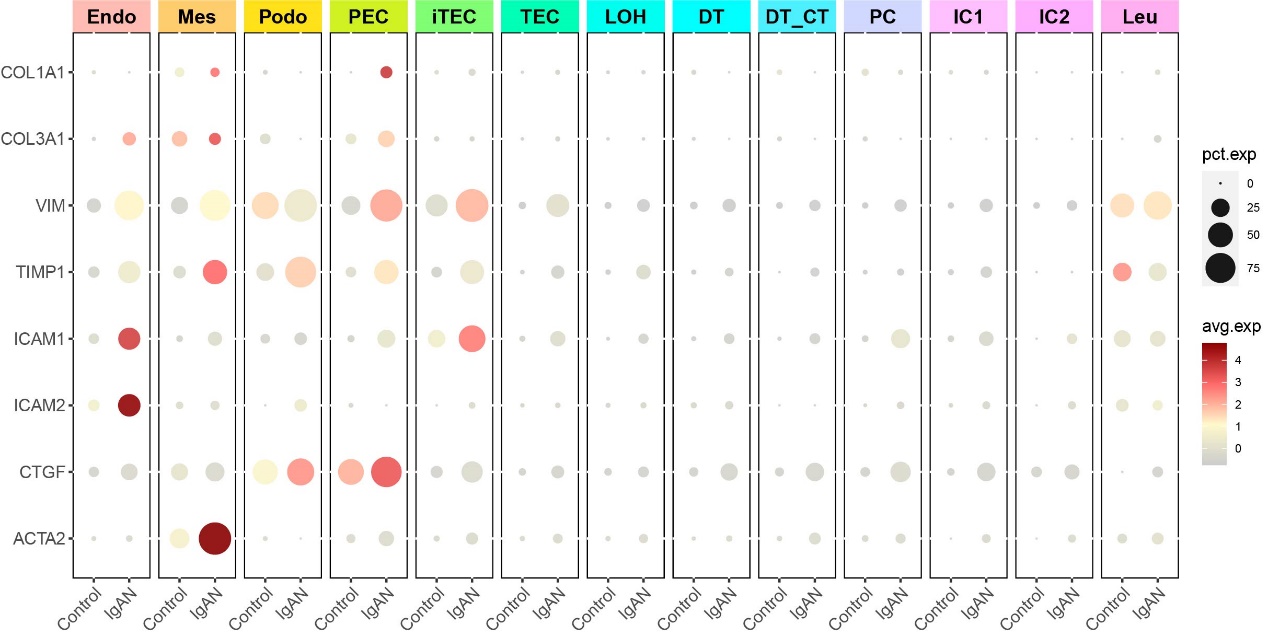


Figure S3: Dotplot showing the fibrosis marker genes expression in cell types, derived from IgAN or healthy control.


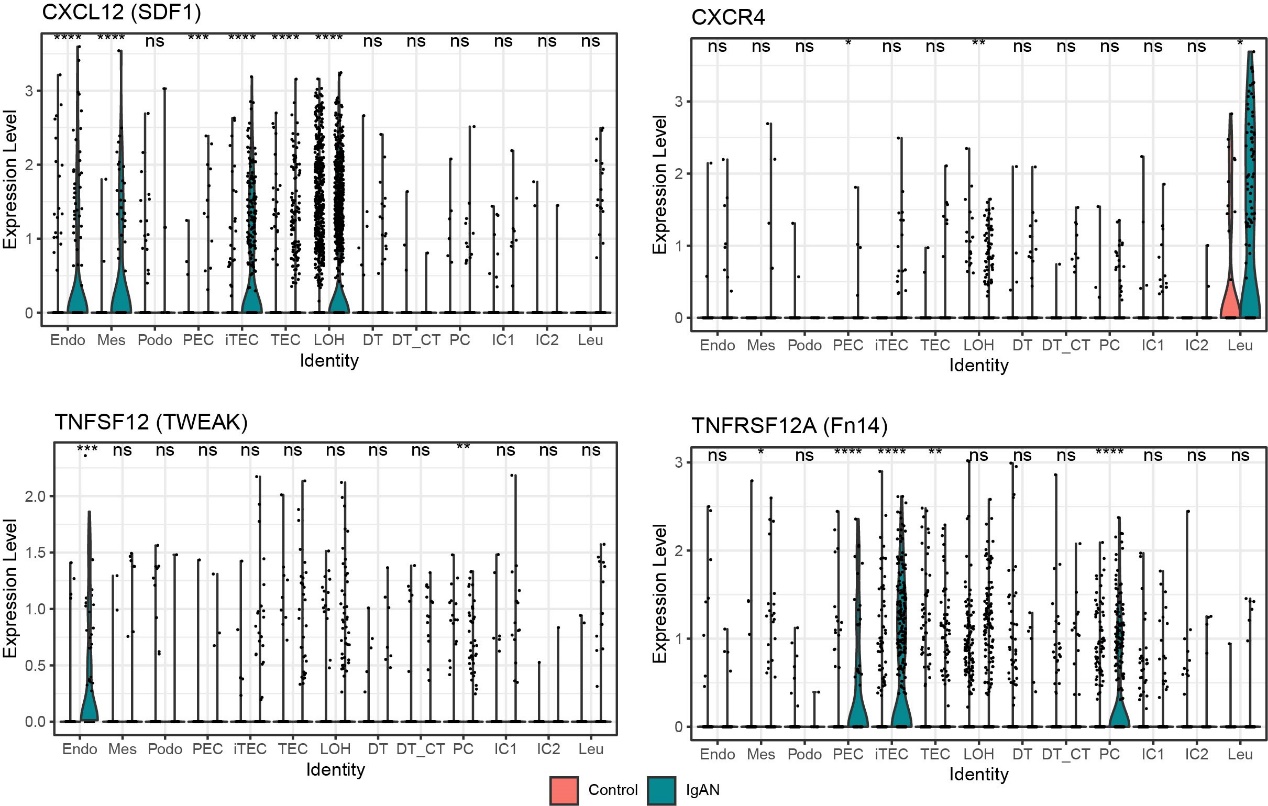


Figure S4: Violin plot showing the expression of inflammatory pathways CXCL12-CXCR4 and TNFSF12-TNFRSF12A in cell types, derived from IgAN or healthy control.


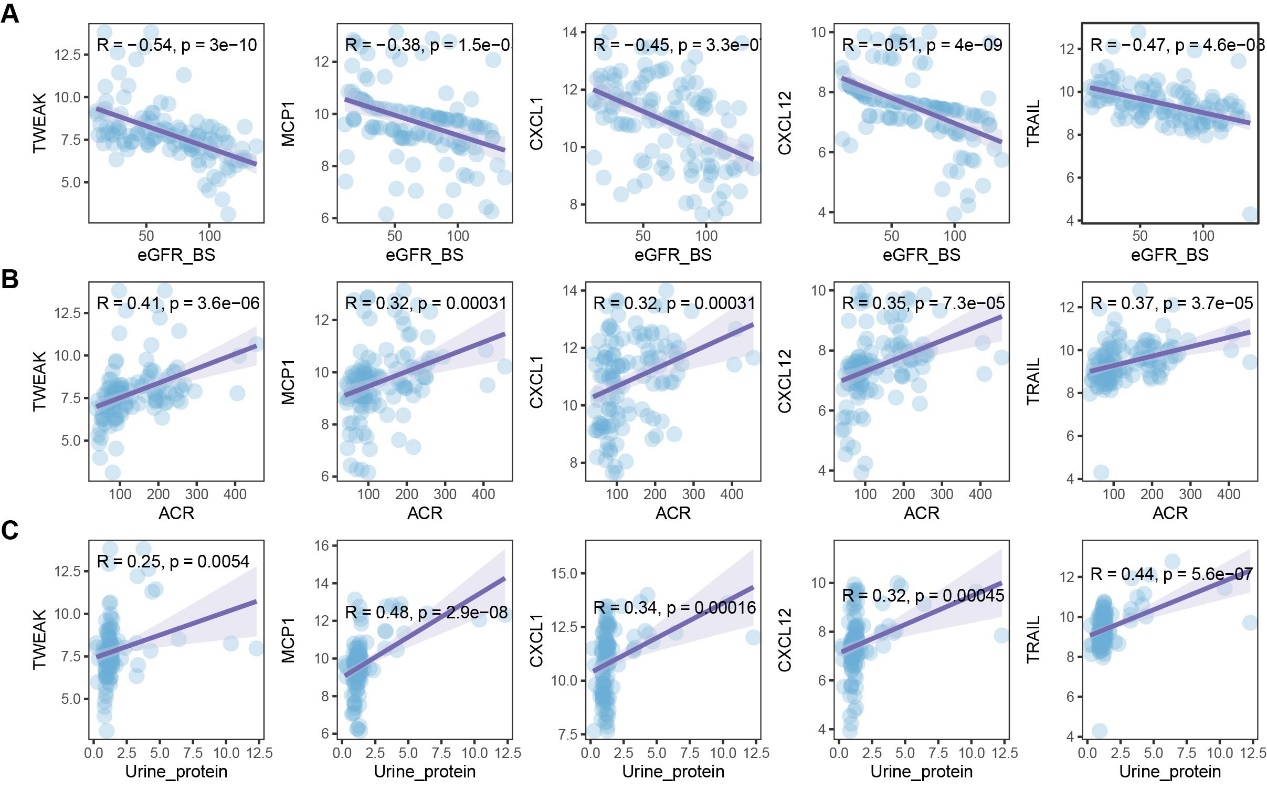


Figure S5: The scatterplot showing the correlations of the urine proteins CXCL1, CXCL12, TWEAK, TRAIL, and MCP1 with the ACR and 24h urine protein value.


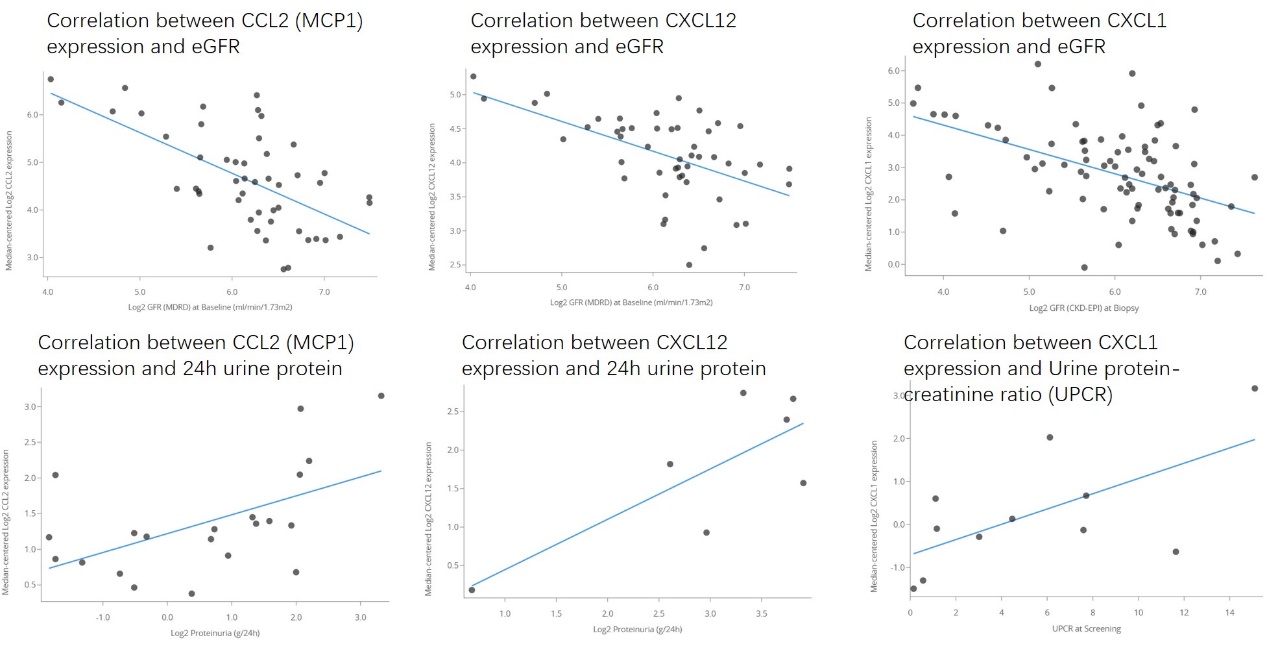


Figure S6: The correlation between urine cytokines biomarkers (CXCL12, MCP1, CXCL1) and the eGFR value, 24h urine protein, and urine protein-creatinine ration.
